# The psychometric performance of generic preference-based measures for patients with pressure ulcers

**DOI:** 10.1186/s12955-015-0307-4

**Published:** 2015-08-01

**Authors:** Simon Palfreyman, Brendan Mulhern

**Affiliations:** Sheffield Teaching Hospitals NHS Foundation Trust, Sheffield, S5 7AU UK; School of Health and Related Research (ScHARR), University of Sheffield, Regent Court, Sheffield, S1 4DA UK; Centre for Health Economics Research and Evaluation, University of Technology Sydney, 1-59 Quay Street, Haymarket, Sydney, NSW2000 Australia

**Keywords:** EQ-5D, SF-6D, Health related quality of life, Psychometrics

## Abstract

**Background:**

Pressure ulcers are wounds that result from reduced mobility, and can have a significant impact on morbidity, mortality and quality of life. As pressure ulcers are a consequence of a wide range of conditions and interventions, it is unclear whether the best means of capturing the quality of life impacts is via generic or condition specific Patient Reported Outcome Measures (PROMs). The aim of this study was to investigate the psychometric performance of the generic EQ-5D and SF-6D amongst patients identified as having or being at risk of developing pressure ulceration.

**Methods:**

A survey of patients who were using pressure relieving mattresses and other equipment was undertaken within inpatient and community settings using a handheld tablet and postal survey. Data on EQ-5D-3L, SF-12 (used to calculate SF-6D), an EQ-5D dignity bolt-on question, demographic and wound specific questions were collected. Convergent validity was assessed using Spearman’s correlations, and agreement using Bland-Altman plots. Known group validity was assessed by examining whether the instruments discriminated between different pressure ulcer severity groups. Multivariate linear regression was used to examine the impact of a range of pressure ulcer related variables.

**Results:**

The total number of participants was 307, including 273 from the acute setting (52 % response rate) and 41 from the community (32 %). SF-6D and EQ-5D were moderately correlated (0.61), suggesting that both instruments were capturing similar quality of life impacts. Both measures were able to significantly discriminate between groups based on the ulcer grade. Presence of a pressure ulcer and number of comorbidities were significant explanatory variables of EQ-5D and SF-6D score.

**Conclusions:**

The results suggest that generic PROMs can effectively capture the impact of pressure ulcers on quality of life, although there are significant challenges in collecting data from this group of patients related to poor clinical condition and mental capacity. The most effective method for obtaining survey data was through the hand held devices and interviewers.

## Introduction

Interventions to prevent and treat pressure ulcers have been identified as a major cost for healthcare providers [1]. In the UK, it has been estimated that the cost of pressure ulceration to the UK health service could be up to 4 % of total expenditure [2]. The cost of treating pressure ulcers depends on their severity and category, and has been estimated as £1214 for the least severe (Category 1) and £14,108 for the most severe (Category 4) [3]. Despite these costs evidence for the cost effectiveness of treatment and prevention strategies is poor [4].

In order to undertake economic evaluations of treatments and prevention strategies for pressure ulcers one essential component is the ability to evaluate their impact on quality of life. This can be done using preference-based patient reported outcome measures (PROMs) which can be used to derive utility values that, when combined with mortality data, can then be used to generate Quality Adjusted life Years (QALYs) [5]. The UK National Institute for Health and Care Excellence (NICE) currently recommends that a generic preference-based measure (i.e. the EQ-5D) is used to generate the utility values for QALYs. However, there is evidence that generic measures may not be sensitive to the quality of life impacts of all conditions [6]. For some health issues, there are also preference-based condition-specific PROMs available: for example dementia [7], venous ulceration [8] and cancer [9]. However, there are a number of concerns with the use of condition specific preference-based PROMS. Firstly, the utilities derived cannot be easily compared (with each other, and with generic PROMs such as EQ-5D), and secondly the impact of comorbidities and/or side-effects may not incorporated into any assessment of quality of life [10]. Furthermore, condition specific preference based PROMs are not widely accepted by reimbursement agencies such as NICE.

The use of PROMs to evaluate the impact of pressure ulcers has been relatively limited [11]. One study compared SF-36 between patients in the community with and without pressure ulcers. They found that a large proportion of the patients were excluded as they were unable to consent, and there were difficulties completing the questionnaires [12]. Essex et al. [13] found that the SF-36 and EQ-5D were sensitive to the impact of pressure ulceration compared to those with no pressure ulcers within the acute setting, and a cohort study of hospital inpatients [14] also found that there were difficulties in recruiting participants and that there were poor completion rates using the SF-36. A condition-specific pressure ulcer PROM has recently been developed (PuQoL [15]). The PuQoL has a large number of questions (*n* = 82) covering ten scales: pain, exudate, odour, sleep, activities of daily living, emotional wellbeing, self-consciousness and appearance, and social participation. A seven dimension preference-based measure has been developed from the PuQoL [16].

In considering the best means of capturing the impact of pressure ulcers on quality of life there are two key considerations which may impact on the use of condition-specific measures such as the PuQoL.

Firstly, the likely domains of quality of life that will be affected for those with a pressure ulcer. Previous studies have highlighted that pain can be a major impact of having a pressure ulcer [17, 18]. Other studies have also described that having a pressure ulcer can have a psychological impact [19], affect usual activities [18], self-care [20] and social activities [20, 21]. Many of these domains are already incorporated into generic measures. One impact that has not been commonly included within either generic measures or the PuQoL is dignity. Having a pressure ulcer has been highlighted as resulting in a loss of self-perceived dignity [19, 22]. This may be particularly acute in those that have a pressure ulcer in the area of the sacrum [23] and for those patients reaching the end of their lives [24].

The second key issue results from pressure ulcers being a consequence of reduced mobility that maybe caused by a large number of diseases, medical interventions and co-morbidities [25]. Any disease or intervention that results in the patient being immobile or unable to change their own position for long periods puts the patient at risk of developing a pressure ulcer. Examples highlighted include: having surgery [26], dementia/cognitively impairment [27], stroke or neurological disease [28], spinal injury [29], diabetes [30], malnutrition [31] and even faecal incontinence [32, 33]. Therefore there may be the potential for any impact of pressure ulceration to be masked by the diversity and range of potential causes of pressure ulcers.

The variety of possible diseases and patient factors linked to pressure ulceration and the issue of impaired dignity being a major impact may mean that the most effective method of capturing the impact of pressure ulceration would be through the use of a generic rather than a condition-specific tool. In addition, studies that have explored the impact of pressure ulceration have highlighted that the key impacts are related to pain, usual activities, mobility, and social impacts, all of which are covered by generic measures. Other issues are directly related to the consequence of having an open wound–such as exudate and smell–but this may be captured within the wider scope of their influence on perceived dignity.

The aim of this study was to explore the psychometric performance of generic preference based PROMS (EQ-5D and SF-6D) in patients with pressure ulcers, or at risk of developing them, to examine the feasibility of using these measures in this patient group.

## Methods

### Data

Patients in the acute and community setting who either had an existing pressure ulcer, or were at risk of developing a pressure ulcer, were identified through a database of patients currently in receipt of pressure relieving mattresses and equipment. Two different data collection methods were used. In the acute setting a handheld tablet device was used to administer the questionnaire and if requested assistance was provided. Due to resource restrictions this was not feasible in the community so a postal survey was carried out. Both surveys consisted of the EQ-5D-3 L [34] and SF-12 [35], demographic and wound specific questions and the Sheffield EQ-5D dignity bolt-on question [36, 37].

Within the acute setting data were collected between May and July 2014. Patients were included if they were currently using a pressure relieving mattress. Information was collected on the total number of patients who were using these types of mattresses in the area, and clinical staff was asked to identify suitable patients. An information leaflet was provided to the potential participant and they were given time to reflect on participation. Verbal confirmation of the presence and category of ulcer was obtained from clinical staff.

The community survey took place in July 2014. Patients were identified from a locally held database of existing users of pressure relieving equipment. A letter was sent detailing the study, along with the questionnaire and a stamped addressed return envelope. Due to the inability to determine the severity of the clinical condition of the recipients only one mail shot was undertaken. It was also not possible to determine the category of the ulcer but this was inferred from asking regarding the presence or absence of a wound, and whether the wound was described as a cavity. If there was no wound it was assumed that there was a category 1 ulcer and if it was described as a cavity it was assumed to be a category 3 or greater. This was felt to be justifiable based on the high likelihood that this at risk population would have category 1 pressure damage indicated by non-blanching erythema. The underreporting of category 1 ulcers and the difficulties and omission of these ulcers from epidemiological and other studies has been highlighted by previous authors [38, 39].

### Measures

#### EQ-5D

The EQ-5D covers five domains of health: mobility, self-care, usual activities, pain/discomfort and anxiety/depression, each with three response levels. The descriptive system describes 243 potential health states, and selection of which were valued using the Time Trade Off preference elicitation method by the UK general population [40]. This resulted in a utility scale (anchored on the full health (1) to dead (0) utility scale) ranging from −0.594 to 1.0 (where states below zero are valued as worse than dead). The EQ-5D is the most widely used generic preference based measure, and utility scales have been developed around the world [41].

#### Sheffield Dignity EQ-5D ‘bolt-on’ question

The Sheffield Dignity Question [36] consists of one dimension with three levels assessing the extent to which respondents feel like they live with dignity that was developed as a ‘bolt-on’ for the EQ-5D. Preference-weights for the EQ-5D plus Sheffield Dignity Question have recently been estimated using DCE incorporating duration (see [42, 43]) resulting in a utility scale ranging from −1.55 to +1.

#### SF-6D

The SF-6D is another widely used generic preference based measure developed by Brazier et al. [44, 45] from the SF-36 [46] and SF-12 [35]. The SF-6D covers six domains of health: physical functioning, social functioning, role limitations, pain, mental health and vitality, with between four and six response levels. A selection of the 18,000 health states were valued using the preference elicitation technique Standard Gamble to produce a utility scale ranging from 0.291 to 1.0.

### Statistical analysis

The psychometric validity of the EQ-5D and SF-6D was assessed in comparison to each other and clinical indicators using standard criteria. There is no gold standard for the assessment of validity, so therefore the validity of measures are assessed in relation to each other, and guided by clinically specific indicators. Statistical analyses were performed using Stata version 13 and SPSS version 21.

### Descriptive analysis

Descriptive EQ-5D and SF-6D scores were examined overall and by pressure ulcer category. The distribution of the responses across the dimensions for the measures was examined overall and by comorbidity. Floor and ceiling effects were assessed by calculating the percentage of patients in the best and worst health states described by the EQ-5D and SF-6D. A large ceiling effect suggests that the measure may not be able to detect improvement in health, and a large floor effect suggests that deterioration in health cannot be captured.

### Convergent validity

Correlations based on the severity of the pressure ulcer and number of comorbidities were calculated between the utility scores, and also between the domains of the instruments using Spearman correlations. Strong correlations indicate that the preference-based PROMs measure similar constructs. Correlations <0.3 are considered weak, ≥0.3 and <0.7 moderate, and ≥0.7 strong.

### Known group validity

Known group validity was assessed by examining whether the instruments were able to discriminate between those who had a pressure ulcer and those who had no wound but were at risk of pressure ulceration. This was done using one-way ANOVA and effect sizes across the sub-groups (calculated as the difference in mean scores between two severity sub-groups divided by the standard deviation of the milder sub-group). Effect sizes of less than 0.2 are small, 0.5 moderate, and 0.8 large [47].

### Agreement

Agreement between the EQ-5D and SF-6D were assessed using a Bland-Altman plot [48]. These show a visual representation of the difference between the scores for the measures by plotting the mean and the difference between the scores. The agreement between the EQ-5D and the SF-6D can also be seen across the range of severity of ulceration through including the upper and lower boundaries around the mean which equate to plus or minus two standard deviations away from the mean difference. In addition, the intra-class correlation coefficients (ICC) were calculated.

### Regression analysis

Linear regression was used to understand the impact of a range of pressure ulcer related variables (including comorbidities and presence of a pressure ulcer) and sociodemographic characteristics on EQ-5D and SF-6D utility score, with both the acute and community sample data included in the model. The regression takes the form:

$$ \mathrm{y} = \mathrm{X}\beta +\varepsilon, $$

where y is the utility score, X represents the explanatory variables, and e represents the error term capturing other explanatory factors. Two models were tested, regressing age, comorbidity prevalence, pressure ulcer presence and the Sheffield Dignity question onto the EQ-5D (Model 1) and SF-6D (Model 2) utility scores.

## Results

### Sample

During the survey a total of 525 patients were screened for inclusion within the acute setting. A total of 212 (52 %) of the patients were unable to be approached to participate in the survey as clinical staff considered that it would be inappropriate. The reasons for this were that the patients had limitations with mental capacity or poor prognosis or were too ill to be approached. This meant that 313 patients were approached for interview and 40 declined to be interviewed. A total of 273 participants were therefore included within the acute setting.

In the community 130 questionnaires were sent and 41 replies received (32 % response rate). However, only 34 responses contained sufficient data to be included in the analysis. This was because four were returned blank or only partially completed and three contained letters detailing that the potential participant had died. A total of 34 participants were therefore included from the Community setting. Therefore the total number of participants in the survey was 307 individuals. The details of the participants are shown in Table 1. The respondents were mostly white British (93 %) with a median age of 77 (range 26–97).

**Table 1:** Demographic details of participants

	Acute	Community	Overall
Age (median)	77	74	
Range	(38–97)	(26–95)	(26–97)
Gender:			
Female (%)	56	78	58
Marital status			
Not stated	12 (4 %)	-	12 (4 %)
Married/civil partnership	108 (40 %)	13 (41 %)	121 (40 %)
Divorced	28 (10 %)	4 (13 %)	32 (10 %)
Widowed	101 (37 %)	12 (30 %)	113 (37 %)
Never married	24 (9 %)	3 (9 %)	27 (9 %)
Comorbidities			
Breathing problems	121 (41.4 %)	8 (25 %)	129 (44.2 %)
Heart problems	61 (20.9 %)	4 (12.5 %)	65 (22.3 %)
Stroke	19 (6.5 %)	7 (21.9 %)	26 (8.9 %)
MS or similar	6 (2.1 %)	17 (53.1 %)	23 (7.9 %)
Spinal injury	16 (5.5 %)	2 (6.3 %)	18 (6.2 %)
Diabetes	66 (22.6 %)	2 (6.3 %)	68 (23.3 %)
Cancer	21 (7.2 %)	4 (12.5 %)	25 (8.6 %)
Fatigue	33 (11.3 %)	11 (34.4 %)	44 (15.1 %)
Depression	20 (6.8 %)	9 (28.1 %)	29 (9.9 %)
Other	69 (23.6 %)	11 (34.4 %)	80 (27.4 %)
Pressure damage			
Pressure ulcer wound	108	28	136
Unable to determine/No PU damage	52	15	67
Category 1 ulcer	87	0	87
Category 2 ulcer	104	14	127
3 or greater	4	4	8
EQ-5D			
Mean (SD)	0.273 (0.38)	0.005 (0.25)	0.243 (0.38)
Range	−0.594–1.0	−0.594–0.71	−0.594–1.0
SF-6D			
Mean (SD)	0.581 (0.10)	0.547 (0.10)	0.578 (0.10)
Range	0.345–0.937	0.34–0.74	0.345–0.937

### Acceptability

The acceptability of the EQ-5D and SF-12 questions as measured by the completion rates of the questions were highest for the Euroqol questions (range 97–98 %), slightly less for the Dignity questions (96 %) and lowest for the SF-12 questions (range 94–96 %). The question that was left blank (94 %) the most often was the question on the SF-12 asking how much physical and emotional health had interfered with social activities. There were no significant floor or ceiling effects present in either of the two instruments (see Table 2). However, there were significant numbers of respondents expressing severe problems within the dimensions in the SF-6D for physical functioning, role limitations and usual activities; and the EQ-5D usual activities.

**Table 2:** EQ-5D and SF-6D floor and ceiling effects

EQ-5D			SF-6D		
	% Reporting no problem	% Reporting severe problem		% Reporting no problem	% Reporting severe problems
Mobility	5.6	38.4	Physical functioning	5.4	58.3
Self-care	21.0	25.3	Social functioning	3.5	25.8
Usual activities	9.5	46.7	Role limitations	4.1	49.3
Pain/discomfort	29.5	12.8	Pain	20.0	8.0
Anxiety/depression	36.5	11.0	Mental health	15.8	2.4
Dignity	44.5	17.4	Vitality	0.7	36.7

### Convergent validity

The mean utility scores for the SF-6D and EQ-5D were significantly correlated with a correlation coefficient of 0.61, suggesting that both instruments are capturing similar impacts of pressure ulceration. However the dimension level correlations were lower, even where larger correlations may be expected (for example between the mental health dimensions). Table 3 includes the correlation coefficients with those expected to be related highlighted in bold.

**Table 3:** Convergent validity of EQ-5D and SF-6D

SF-6D	EQ-5D					
	Mobility	Self-care	Usual Activ	Pain	Anxiety	Dignity
PF	0.34	0.37	0.43	0.24	0.29	0.31
RL	0.26	0.29	0.24	0.15	0.39	0.24
SF	0.31	0.25	0.37	0.09	0.17	0.28
PN	0.20	0.14	0.17	0.54	0.24	0.23
MH	0.25	0.19	0.18	0.25	0.24	0.23
VT	0.18	0.29	0.35	0.18	0.21	0.29

*PF* Physical Functioning, *RL* Role Limitations, *SF* Social Functioning, *PN* Pain, *MH* Mental Health, *VT* Vitality

### Known group validity

One-way ANOVA found that EQ-5D and SF-6D were able to significantly discriminate between those that had an ulcer and those who were at risk but currently had no ulcer. The difference between the mean EQ-5D (*p* = 0.01) and SF-6D (*p* = 0.023) scores was significant based on the ulcer category. The effect size based on presence or absence of ulceration was 0.4 for the EQ-5D and 0.3 for the SF-6D. The instruments had some success in discriminating on the basis of comorbidities and presence of an ulcer (see Table 4). A comparison of the mean scores based on the severity of the ulcer i.e. category and also on presence or absence of an ulcer showed a clear decrement for both the SF-6D and EQ-5D based on the presence/absence of an ulcer increase in category of ulcer showed a (see Table 4) . The difference in mean SF-6D scores was significant using on a one-way ANOVA for the both ulcer category (*p* = 0.024) and presence of an ulcer (*p* < 0.001). The difference in mean scores for the EQ-5D was even clearer for both ulcer category (*p* = 0.001) and ulceration (*p* < 0.001).

**Table 4:** EQ-5D and SF-6D known group validity

	EQ-5D				SF-6D			
	N	M (SD)	ES	Sig	N	M (SD)	ES	Sig
Category				0.001				0.02
0	87	0.364 (0.36)			84	0.602 (0.11)		
1	77	0.250 (0.36)	0.32		76	0.580 (0.07)	0.20	
2	122	0.160 (0.38)	0.25		117	0.561 (0.10)	0.27	
3/4	8	0.115 (0.34)	0.12		8	0.536 (0.10)	0.25	
Ulcer status				0.005				0.008
No wound	160	0.310 (0.36)			160	0.592 (0.10)		
Current ulcer	130	0.160 (0.38)	0.40		125	0.560 (0.10)	0.32	
N of comorbidities				0.001				*P* < 0.001
0		0.397 (0.35)				0.631 (0.13)		
1		0.196 (0.37)	0.57			0.578 (0.10)	0.41	
2		0.229 (0.38)	0.08			0.575 (0.10)	0.03	
3		0.257 (0.36)	0.07			0.548 (0.08)	0.27	
4		−0.030 (0.23)	0.79			0.502 (0.12)	0.58	
5+		−0.024 (0.23)	−0.02			0.438 (0.75)	0.09	

### Agreement between measures

**Fig. 1:**
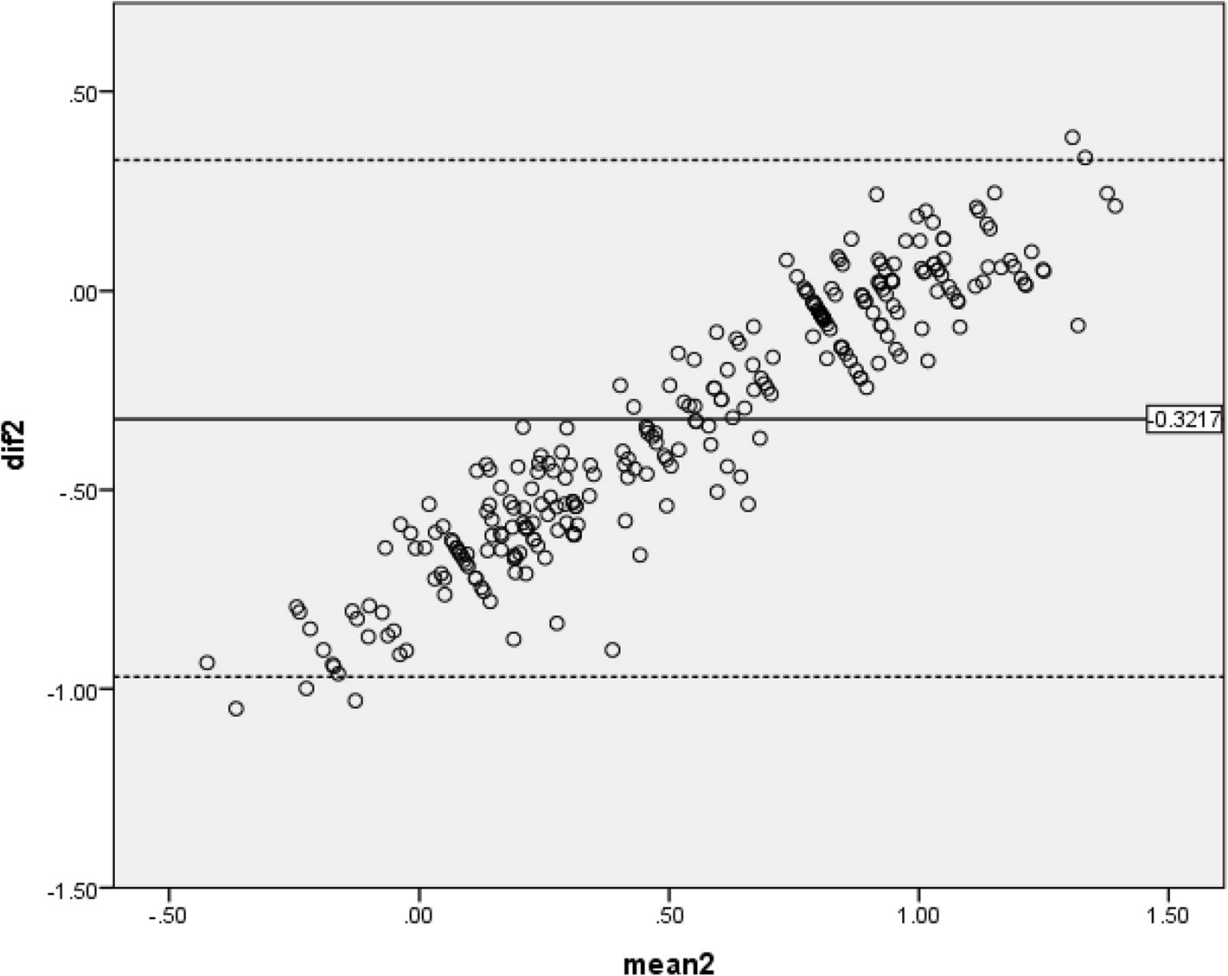
Agreement between EQ-5D and SF-6D

The Bland Altman plot shown in Fig. 1 illustrated that agreement between the measures was highest at the lower end of the scale where there were lower levels of quality of life (i.e. the mean of the measures is lower.

### Regression analysis

The regression reported in Table 5 demonstrates that number of comorbidities significantly explained EQ-5D and SF-6D utility score, with lower utilities reported as the number of comorbidities increases. Presence of a pressure ulcer is also a significant explanatory variable, with the negative coefficient indicating that not having a pressure ulcer is linked to a better EQ-5D or SF-6D score. The size of the coefficients is in line with the difference in utility scores between those with and without a pressure ulcer reported in the known group validity analysis in Table 4, and is larger for EQ-5D given that the utility scale covers a larger range than the SF-6D (and this is the case for all of the parameters included in the model). The Sheffield dignity question was also a significant explanatory variable, where a lower level of dignity as self-reported by this question was linked to a lower utility score. Age was not a significant parameter, but this could be linked to the skewed nature of data, with the vast majority at the upper end of the age range.

**Table 5:** Regression results

Parameter	EQ-5D		SF-6D	
	Coeff.	Sig.	Coeff.	Sig.
Age	−0.008	0.721	0.008	0.186
Total co-morbidities (Baseline = 0)				
1	−0.198	0.000	−0.043	0.003
2	−0.137	0.012	−0.040	0.006
3	−0.084	0.191	−0.060	0.001
4+	−0.276	0.002	−0.106	0.000
Current pressure ulcer	−0.156	0.000	−0.034	0.002
EQ-5D dignity question	−0.227	0.000	−0.058	0.000
N	291		284	
R squared	0.30		0.30	
RMSE	0.30		0.09	

*RMSE* Root mean squared error

## Discussion

It is well established that pressure ulcers have a significant impact on quality of life [21]. In recent years, research has attempted to establish the best method to capture the impact on quality of life to better evaluate the clinical and cost effectiveness of interventions for the treatment and prevention of pressure ulcers. Pressure ulcers are a consequence of reduced mobility which may result from a wide range of both acute or chronic illness and treatment. The significance of this is that there are likely to be difficulties in separating the impact of the cause from the consequence (i.e. pressure ulcer) of the illness, and therefore generic measures which allow for the wider assessment of the HRQL impacts of conditions, may prove useful. Pressure ulcers are a consequence of being immobile or having reduced mobility. Therefore by definition anyone who has an intervention, disease or co-morbidity that affects mobility will be at risk of developing a pressure ulcer. The potential number of interventions and diseases may mean that having one condition-specific instrument able to capture the impact for this variety would be difficult. The development and use of PROMs specifically tailored to measure clinical consequences of a condition, such as pressure ulcers, may therefore be problematic: in the case of pressure ulceration has been described as “difficult, if not impossible” [20].

In the current study both the generic measures (EQ-5D and SF-6D) showed the ability to discriminate between those who were considered to be at risk of developing and those who had a wound, and also based on the severity of pressure ulcer. The presence of a category 1 to ≥3 as assessed for the acute population also led to a significant deterioration in score for both measures. These results suggest that generic PROMs can effectively capture the impact of pressure ulcers on quality of life, and therefore have some level of validity for use in groups of patients with pressure ulcers in both the acute and community sector. The results were in line with other work testing generic (EQ-5D and condition specific (PuQOL-UI) utilities in pressure ulcer patients [16]. Given the ease of completion of the EQ-5D and SF-12, the ability of generic measures to directly compare across conditions, and their likely sensitivity to the impacts of the underlying health condition leading to the pressure ulcer, generic measures can be used with some level of confidence in pressure ulcer patient groups.

The convergent validity results show that the utility scales are moderately correlated, but there are lower correlations between the descriptive systems, even across dimensions where a higher correlation would be expected (for example EQ-5D mobility and SF-6D physical functioning). This has been demonstrated in other health areas [43] and suggests that although the instruments are measuring some overlapping constructs, there are divergences that may be somewhat masked by the complementary nature of the utility scales.

The current study highlighted that collecting data from patients who have developed, or are at risk of developing, pressure ulcers can be achieved most effectively within the acute setting rather than the community. The large number of patients who were unsuitable or unable to participate in the study due to poor cognition and clinical condition also suggested that administration should be guided by interviewers rather than relying on self-completion. This has potential resource issues due to the increased cost of interviewer guided compared to postal administration. The difficulties of obtaining data on pressure ulcers within the community setting, due to the complexity and variety of localities, has also been highlighted previously [49]. It is also likely to be compounded by the nature of the population who develop pressure ulcers who tend to be elderly, in poor health and have potential cognitive impairment [50, 51]. Within the current study more than half the potential participants could not be approached due to problems with cognition or their clinical condition being too severe. In those who completed the survey 78 % had one or more comorbidities, 49 % had two or more, and 21 % had three or more comorbidities. The impact of an ill population and the selection bias imposed by only those able to answer questions would likely be even more of an issue in the community. However, data was successfully collected using both handheld devices and postal methods, and combining data collection modes may increase response rates provided that score equivalence can be demonstrated. There is evidence for this for the newly developed five level EQ-5D [43]. The recently developed EQ-5D-5 L may also show improvements in sensitivity for this group of patients.

This study has highlighted the complexity of those who develop pressure ulcers. It also emphasised that these wounds are a consequence of a range of conditions and interventions which result in reduced mobility. One consequence of this may be that a range of PROMs would need to be constructed to be sensitive to all of the HRQL impacts. However, the current study highlighted that existing generic measures seem to incorporate many of the important impacts of pressure ulceration. It may therefore be most appropriate to use generic rather than condition-specific PROMs when conducting economic evaluations of interventions aimed at preventing and treating pressure ulcers.

### Limitations

The responsiveness of the questionnaires to improvements in the condition of the pressure ulcer were not evaluated due to resource limitations. However, for this population particularly those with the most severe categories the patient’s clinical condition may remain poor. Due to the nature of the survey methods no physical assessment of the participants were possible. Participants were therefore providing self-reported presence of skin damage and so it was not possible to determine category of pressure ulcer. However, the population were all at risk of pressure ulceration as they were using pressure relieving equipment and it would be reasonable to infer the more severe categories on the basis of questions asking about cavity, exudate and smell.

## Abbreviations

EQ-5D: EuroQol 5-dimension
MH: Mental health
NICE: National Institute for Health and Care Excellence
PF: Physical functioning
PN: Pain
PROMS: Patient reported outcome measures
PuQoL: Pressure ulcer quality of life
QALY: Quality adjusted life year
RL: Role limitations
RMSE: Root mean squared error
SF: Social functioning
SF-6D: Short form-6 dimensions
SF-12: Short form-12
SF-36: Short form-3
VT: Vitality
